# Development of a new measure for mental retirement; testing of a three-factor structure of mentale retirement in different subgroups

**DOI:** 10.1186/s12889-019-7649-5

**Published:** 2019-12-02

**Authors:** Jenny J. J. M. Huijs, Irene L. D. Houtman, Roland W. B. Blonk

**Affiliations:** 1 TNO (The Netherlands Organization for Applied Scientific Research) & Utrecht University (Dept. of Social, Health and Organizational Psychology), Leiden, Netherlands; 2 TNO (The Netherlands Organization for Applied Scientific Research), Leiden, Netherlands; 3 TNO (The Netherlands Organization for Applied Scientific Research) & Tilburg School of Social and Behavioral Sciences (Dept. of Human Resource Studies and Dept. Tranzo), Leiden, Netherlands

**Keywords:** Mental retirement, Employability, Development, Engagement, Appreciation, Construct validity

## Abstract

**Background:**

The aim of this study is to develop a new measure for the concept of mental retirement and test the construct validity of the measure. Employees who are ‘mentally retired’ are present at their work physically, but have already said their goodbyes mentally. Mental retirement has a three-factor structure: developmental proactivity, work engagement and perceived appreciation.

**Methods:**

We use data from employees (*N* = 867) of five different organizations in the Netherlands. Mental retirement was assessed with 11 items in an online survey. In addition, socio-demographic characteristics like age, level of education and occupation, were measured. Next to tests of internal consistency, a confirmatory factor analysis (CFA) is performed to test the three-factor structure of mental retirement in this population and in different subgroups (age, education, occupation).

**Results:**

The internal consistency varies from .80 to .94 for the developmental proactivity scale and the work engagement scale, respectively (appreciation was measured with one item). For the CFA, the three-factor model fits the data adequately. Multiple group analyses also shows equal factor loadings in all subgroups, but the mean levels of mental retirement differ across subgroups.

**Conclusions:**

This study confirms the three-factor model of mental retirement in a general group of employees as well as across different subgroups. However, this study only tested the construct validity. Future research should study validity more extensively and be longitudinal in nature. In addition, the causal chain of antecedent variables to mental retirement and its outcomes should be considered. These studies could also focus on the effects of interventions aiming at preventing or decreasing the level of mental retirement in organizations.

## Background

“Why should I care? They keep on making changes, but it doesn’t make any difference”. Or “That new colleague is so annoying, all day every day he comes up with new ideas and methods. Let me just do my work and don’t bother me”. Almost everybody will know a colleague who acts this way. Someone who seems to be disconnected from his work and the organization. A colleague who seems to be counting the hours of each working day and the days until retirement and whose motivation to work has gradually shifted from intrinsic to instrumental. ‘My job’ and ‘my company’ have become ‘that job’ and ‘that company’. Such employees may be described as mentally retired from the organization, while they remain working. In this paper we will define the concept of mental retirement and present a new measure for mental retirement.

Employees who are ‘mentally retired’ are present at their work physically, but have already said their goodbyes mentally. They appear to invest less in their work, in their employability and development, they don’t want to walk the extra mile anymore and have gradually lost their connection to their job, their colleagues and the organization.

Based on an initial Dutch exploration consisting of a literature search, expert meetings, focus groups as well as previously collected data, mental retirement appears to consists of three aspects [1]. Firstly, a decline in interest in learning and developing appears to be an important characteristic of mental retirement. The lack in development may lead to a decline in or even loss of skills, in skills obsolescence, in a decrease in sustainable employment for both the internal labor market of an organization as well as the external labor market or even in job loss [2–4].

Secondly, reduced engagement or reduced motivation appears to be a key aspect of mental retirement. Employees who are less engaged or even burn-out show low levels of energy and poor identification with one’s work and low job performance [5, 6]. Less work engagement is also related to more sickness absence [7]. This implies that if employees are less engaged, they have a higher risk to lose the connection to their work and their colleagues and therefore have a higher risk to become mentally retired.

The third and final aspect of mental retirement is linked to appreciation. Employees who are ‘mentally retired’ will perceive themselves and their work as less valued appreciated by either, colleagues, supervisor or the organization as a whole. Employees who perceive more appreciation and have more meaningful work seem to be more committed to their job [8, 9].

This new concept of mental retirement appears to be closely related to burnout. Burnout has three core dimensions: exhaustion, cynicism (or depersonalization) and reduced professional efficacy [10]. Distancing oneself or pulling away from work is a key aspect of mental retirement, which corresponds with the cynicism or depersonalization dimension of burnout. However, in mental retirement another key aspect is a lack of interest in learning and development. Another distinction between mental retirement and burnout is that exhaustion is often seen as the most prominent aspect of burnout, while employees who are mentally retired are not so much exhausted but rather indifferent [10].

In addition, in the literature the concept of mental retirement has been mentioned before [e.g. 11,12]. In these earlier studies mental retirement has a definition which is specifically linked to age. Mental retirement is defined for example as the cognitive decline that appears to occur after actual retirement [11]. This decline is caused by a lack of cognitive stimulation and mental exercise, which arises when someone actually retires as well as when an employee is still working but has the prospect of nearby retirement. In another study mental retirement is defined as a decrease in work engagement for employees who are facing actual retirement [12].

Another related concept is the ‘older worker identity’. This refers to the internalization of negative attitudes and beliefs regarding the older worker, mostly based on stereotypes (e.g. low motivation, resistance to change, inflexibility and lack of interest in learning) [13, 14]. This internalization can be caused by discrimination in career opportunities and feelings of deprivation in comparison to others.

Both the concept of older worker identity and the definitions mentioned earlier on mental retirement are linked to age. Although studies show that older employees participate to a lesser degree in training and maintenance of their working skills [15–18], a study on lifelong learning in the Netherlands shows that there may be a trend shift over time with regard to training participation [19, 20]. In the past, training participation clearly declined with age, but data from 2010 indicates that training participation remains stable. In addition, studies have shown that the importance of meaningful work, development opportunities and being appreciated increases with age of retirement [21, 22]. Thus, we are not convinced mental retirement is linked to older age. Therefore, in this study we examine the link between older age and mental retirement in order to get better insight in this relation.

We expect that mental retirement may have many negative consequences for employees (employability, job satisfaction, fulfillment etc.), and also for the organization (reduced productivity, quality of work) and the society in general (costs due to early retirement, well-being or even unemployment in the long run). The concept of mental retirement may become more important since sustainable employability is becoming a more essential and important topic for individuals, organizations as well as society. Therefore it is important to understand this new concept of mental retirement better and develop a good measure for mental retirement. In this paper we aim to develop this new measure of mental retirement and test its construct validity. Furthermore, we consider the discriminant validity across different groups of employees. Regarding the latter, as discussed before age may be such a differentiating employee characteristic. The level of mental retirement may be higher in older employees since literature shows that older employees are less likely to participate in vocational training or on-the-job training, maintain their working skills, are also less supported and encouraged to engage in learning activities and have a higher chance the become disengaged from work [15–18, 23]. On the other hand, the importance of meaningful work, development opportunities and being appreciated increases with age of retirement [21, 22].

Next to age, the level of mental retirement may differ between employees with different levels of education as well. Lower educated employees participate less often in training than higher educated employees and this training gap has grown in recent years [20]. In addition, they are more at risk for low work engagement or high levels of emotional exhaustion [24, 25]. Therefore, we expect a higher level of mental retirement among employees that are less educated.

Besides age and level of education, occupation may play a role as well. Research shows that employees in some occupations, e.g. police officers or nurses, have a higher level of engagement and may also be more intrinsically motivated than employees in other occupations, e.g. blue-collar workers [26–28]. In comparison with other public service employees, police officers feel appreciated and are not unsatisfied with their own organization [29]. So, the level of mental retirement is expected to be lower for service workers, such as police officers, as compared to other occupational groups like office workers.

The current study aims to develop a new measure for mental retirement and test if mental retirement consists of three factors: developmental proactivity, work engagement and perceived appreciation.

- Hypothesis 1: the three-factor model will fit a general group of employees with a broad distribution of age, education and occupation.

In addition, we examine if the three-factor structure is stable across different subgroups.

- Hypothesis 2: the three-factor structure will be invariant across subgroups (which differ in age, education and occupation).

However, we expect the mean levels of mental retirement to differ across these subgroups:

- Hypothesis 3: the level of mental retirement is higher in older employees (> = 50 years) as compared to younger employees.
- Hypothesis 4: the level of mental retirement is higher in less educated employees as compared to higher educated employees.
- Hypothesis 5: employees in office jobs have a higher level of mental retirement than service workers like police officers.

## Methods

### Design and procedure

The research population of this study consisted of a sample of employees of five different public and private organizations in the Netherlands. Three departments of the National Police participated: two departments of police officers and one facility department (resp. *N* = 175; *N* = 185; N = 175). The other four organizations were an archive department of the government (*N* = 291), a department of the Dutch Ministry of Education that provides student financing (*N* = 233), ten teams from an organization that implements national insurance schemes in the Netherlands (*N* = 209) and one team of management assistants of a health technology organization (*N* = 63).

Online questionnaires were send to every employee of the different organizations (*N* = 1331). The questionnaires were send between March 2014 and October 2015 (depending on when the organization started with the project). These questionnaires were part of a bigger, associated, program that was introduced in the five organizations.

### Measures

Mental retirement was measured with three concepts (see Appendix 1 for the questionnaire). Firstly, *developmental proactivity* consisted of four items [30]. The response categories ranged from 1 (“totally disagree”) to 5 (“totally agree”). Secondly, six items tapped *work engage*ment (derived from the Utrecht Work Engagement Scale (UWES) [26]. Respondents were asked to describe how often they experienced the described situations (1 = never; 7 = always). Thirdly, *perceived appreciation*, is measured with one question. The response categories ranged from 1 (“not at all”) to 4 (“very much”).

Seven single items measured socio-demographic characteristics (gender, age, level of education, occupation, number of working hours according to contract, years working in the organization and in the function).

### Statistical analysis

Fist, differences at baseline between the subgroups were tested with Pearson Chi-square tests and t-tests.

Second, to assess the internal consistency of the three concepts of mental retirement, Cronbach’s alpha was computed for each scale, both in the whole group as well as within each subgroup. Age and occupation were each divided in two subgroups: younger than 50 years; 50 years and older; police officers and office jobs. The facility department of the Dutch police and the teams of the other four organizations can be seen as office jobs. Education was divided in three subgroups: low, intermediate and high level of education.

Third, a confirmatory factor analysis (CFA) with Lavaan, R Package for Structural Equation Modeling [31] was applied to examine the construct validity of mental retirement. Chi-square difference tests (χ2-test) were used to evaluate the relative fit of the three-factor model. A non-significant value indicates a good fit with the data. However, this index is very sensitive to sample size (significance increasing as sample size increases). Hence, additional fit indices were added. The root mean square error of approximation (RMSEA) and standardized root mean square residual (SRMR) avoid problems with sample size. The RMSEA reflects the extent to which the model fits the population covariance matrix, where an acceptable model fit is reflected by values < 0.08 [32]. The SRMR is a standardized summary of the average covariance residuals, and indicates a good fit for values < 0.08 [33]. Goodness of fit was therefore also evaluated by using the Tucker-Lewis index (TLI) and the Comparative Fit Index (CFI). The TLI and CFI indices compare the hypothesized model to a ‘null’ or worst fitting model, taking into account model complexity, and indicate an acceptable model fit for values > 0.90, and good model fit with values > 0.95 [33].

Three multigroup structural equation models were proposed a priori to compare the factor loadings across age subgroups (< 50 years; = > 50 years), educational level (low; intermediate; high level of education) and occupation (police officers; office jobs). In model 1 (baseline model), the factor loadings of the prespecified three-factor model were estimated freely within each subgroup. In model 2 (metric invariance model), the factor loadings were constrained to be equal across the subgroups. Comparison of model 1 to model 2 represents a test of measurement equivalence across subgroups. Differences between the two models are examined with the chi-square difference test and the ΔCFI. Changes in CFI values of 0.01 or less are indicative of factor invariance across the groups [34].

## Results

In total 867 (65%) employees filled out the questionnaire. The mean age of the participants was 46.7 years (see Table 1) and a mall majority were male (54.4%). Most participants worked fulltime (69%) and have been working almost 14 years within the current organization. Table 1 also shows differences in baseline characteristics between the subgroups within the variables age, education and occupation. Employees of 50 years and older and employees who are lower educated are more often male and are working longer in their organization and in their function. Police officers are younger, more often male, lower educated, more often work fulltime or less than 12 h and are working longer in their organization than employees with office jobs.

**Table 1:** Baseline characteristics of the participants

		Total	Age		Education			Occupation	
			< 50 year	> = 50 year	Low	Interme-diate	High	Police officers	Office jobs
	N:	867	449	380	176	408	257	230	637
	%:	100%	54%	46%	21%	49%	31%	27%	73%
Age	Mean	46,7	38,1▼	**56,9▲**	46,3	47,3	45,9	40,4▼	**48,9▲**
	SD	11,1	7,12	4,31	11,7	11,0	11,0	11,1	10,2
Gender	Male	54,4%	49,4%▼	**59,5%▲**	65,9%▲	56,9%	42,4%▼	**70,3%▲**	48,8%▼
	Female	45,6%	**50,6%▲**	40,5%▼	34,1%▼	43,1%	**57,6%▲**	29,7%▼	**51,2%▲**
Education	Lower	20,9%	20,7%	20,1%	100%	–	–	41,6%▲	13,7%▼
	Intermediate	48,5%	46,1%	51,7%	–	100%	–	53,0%	46,9%
	High	30,6%	33,2%	28,2%	–	–	100%	5,5%▼	**39,4%▲**
Working hours per week	> = 35 h	69,0%	70,4%	67,6%	68,2%	69,9%	68,1%	74,9%**▲**	66,9%▼
	20–34 h	28,7%	27,8%	29,7%	27,8%	28,2%	30,4%	21,5%▼	31,3%▲
	12–19 h	1,4%	0,9%	1,8%	2,3%	1,2%	1,2%	0,9%	1,6%
	< 12 h	0,8%	0,9%	0,8%	1,7%	0,7%	0,4%	**2,7%▲**	0,2%▼
Years working at organization	Mean	13,7	10,0▼	**18,1▲**	15,5**▲**	14,5	**11,3▼**	**15,3▲**	13,1▼
	SD	11,3	6,79	13,8	11,9	11,7	10,0	10,9	11,4
Years working in function	Mean	6,26	5,05▼	**7,66▲**	7,13**▲**	6,76**▲**	**4,89▼**	6,48	6,18
	SD	6,43	4,56	7,89	7,36	6,64	5,15	6,59	6,38

▲ and ▼: *p* < 0,05, significant high (low) percentages and/or means.

The internal consistency for the developmental proactivity scale and the work engagement scale was very good (overall and within group are presented in Table 2). The Cronbach’s alfa of the developmental proactivity scale varied between .80 and .88. For work engagement the internal consistency was consistent with .94 in the whole population as well as in every subgroup. Perceived appreciation was measured with one item and therefore no internal consistency could be calculated.

**Table 2:** Cronbach’s alfa for mental retirement

Subgroup	N	Cronbach’s alfa (α) Developmental pro-activity	Cronbach’s alfa (α) Work engagement
Whole group	848–853	.85	.94
Male	451	.85	.94
Female	376–378	.84	.94
< 50 year	444	.82	.94
> = 50 year	372–374	.88	.94
Police officers	223–224	.80	.94
Office jobs	625–629	.86	.94

The three factors of mental retirement are correlated, but they also clearly distinct from each other (see Table 3). Table 3 also displays the scores on mental retirement for the whole group.

**Table 3:** Scores on mental retirement and Pearson correlation (*N* = 848–853)

	Total		Pearson correlations		
	M	SD	Developmental pro-activity	Work engagement	Perceived appreciation
Developmental pro-activity [Range: 1–5]	4,14	0,59	–	.43**	.30**
Work engagement [Range: 1–7]	4,86	1,30		–	.48**
Perceived appreciation [Range: 1–4]	2,51	0,80			–

**p < .01 (2-tailed)

The CFA shows that the three-factor model appeared to fit the data adequately (confirmation hypothesis 1). The Chi-square difference test, however, was significant (see Table 4). This apparent lack of fit is not surprising, because very small differences between expected and observed correlations in large samples can lead to a significant χ2-test [34]. The other goodness-of-fit indices showed good fit (CFI; TLI > .90 and SRMR <.08), except the RMSEA score, which was relatively high with 0.11.

**Table 4:** Goodness-of-fit indices for the mental retirement model (*N* = 853)

Model	χ2	df	CFI	TLI	RMSEA	SRMR
Whole group	484.72	42	.93	.91	.11	.03

Multiple group analyses examined the invariance of the three-factor model across subgroups. All multiple group analyses revealed (see Table 5) that the chi-square tests are not significant and the ΔCFI is smaller than or equal to the proposed cutoff point of .01 [34]. This indicates that the factor loadings can be assumed equal in all subgroups and hypothesis 2 is confirmed.

**Table 5:** Goodness-of-fit indices for the subgroups of the mental retirement model (*N* = 853)

	χ2 (∆χ2)	df (∆df)	p (∆p)	CFI (∆CFI)
Age				
Model 1	584.95	84	<.001	.923
Model 2	(4.81)	(8)	(.78)	(.000)
Education				
Model 1	591.65	126	<.001	.927
Model 2	(21.66)	(16)	(.15)	(.001)
Occupation				
Model 1	556.44	84	<.001	.927
Model 2	(14.54)	(8)	(.07)	(.001)

In addition, the mean scores of mental retirement in the different subgroups were assessed. No differences in levels of developmental proactivity, work engagement and perceived appreciation were found for age, so hypothesis 3 is rejected. However, some differences were found across educational levels. Higher educated employees report higher levels of developmental proactivity and perceived appreciation as can been seen in Table 6. In addition, the scores of work engagement are higher for lower educated employees. Consequently, hypothesis 4 is partly confirmed. Hypothesis 5 is also partly confirmed, since police officers are more engaged than employees with office jobs. But no difference were found in levels of developmental proactivity or perceived appreciation across occupation groups.

**Table 6:** Scores on mental retirement in the subgroups

		Total	Age		Education			Occupation	
			< 50 year	> = 50 year	Low	Intermediate	High	Police officers	Office jobs
	N:	853	444	374	167	404	257	224	629
	%:	100%	54%	46%	20%	49%	31%	26%	74%
Developmental pro-activity Mean [Range: 1–5]	Mean	4,14	4,18	4,10	4,12	4,11	**4,23▲**	4,15	4,14
	SD	0,59	0,56	0,63	0,63	0,59	0,56	0,51	0,62
Work engagement Mean [Range: 1–7]	Mean	4,86	4,82	4,88	**5,55▲**	4,72▼	4,63▼	**5,51▲**	4,63▼
	SD	1,30	1,31	1,30	1,45	1,24	1,15	1,12	1,28
Perceived appreciation Mean [Range: 1–4]	Mean	2,51	2,50	2,51	2,57	**2,39▼**	2,66▲	2,52	2,50
	SD	0,80	0,79	0,83	0,91	0,73	0,81	0,77	0,81

▲ and ▼: *p* < 0,05, significant high (low) means

## Discussion

First of all, the current study tested the structure of the new measure for mental retirement using confirmatory factor analysis. The results of this study on a population which considerably varied in age, educational level and occupational group confirmed the three-factor model of mental retirement which consists of developmental proactivity, work engagement and perceived appreciation. In addition, multiple group analyses showed equal factor loadings in all subgroups implying the three-factor model was stable across subgroups (age (< 50 years; > = 50 years), level of education (low, intermediate, high) and occupation (police officers and office jobs)).

Secondly, it was shown that the mean levels of mental retirement did indeed differ across educational level and occupational group, but not across different age groups. Higher educated employees reported a higher level of developmental proactivity and perceived appreciation, but lower levels of engagement. These differences in developmental proactivity and appreciation may be found because higher educated employees participate more often in training and are more motivated to participate in training activities [20, 35]. However, contrary to our expectations lower educated employees showed higher levels of work engagement than intermediate or higher educated employees. This is not in line with previous research [24, 25]. A possible explanation is that in our sample the group of lower educated employees proportionally includes more police officers and this group is known to report a higher level of work engagement in comparison with employees with office jobs [26, 27, 36]. These findings suggest that the concept of mental retirement is more prevalent for lower educated (as compared to middle and higher educated employees), and that the concept is also more prevalent for office workers as compared to police officers. Although tempting, this study does not generalize the latter finding to all service workers, or to a specific group of (higher educated) service workers.

This study also shows that the concept of mental retirement may be as important in younger employees as in older employees near the end of their work life. Although studies show that older employees participate less in training and maintenance of their working skills [15–18], the level of developmental proactivity doesn’t appear to decrease with age in our study. A study on lifelong learning in the Netherlands shows that there may be a trend shift over time with regard to training participation [19, 20]. In 2004 training participation clearly declined with age, but data from 2010 indicates that training participation remains stable. Our findings also show that the level of work engagement and perceived appreciation may not decrease with age. In our study, mental retirement appears not to be linked with older age making the concept of mental retirement differ from the concept of older worker identity and the other studies that used a different definition of mental retirement [11–14]. In addition, studies also report that age may be important for mental retirement, but should be considered in interaction with job characteristics such as the meaningfulness of the work. The importance of meaningful work, development opportunities and being appreciated have been shown to increase with age of retirement [21, 22]. The impact of these interaction effects may be best studied when including (perceived) job characteristics and outcome variables, such as health outcomes, sickness absence or actual exit from the job. Age may still be important in relation to mental retirement, but should be considered in relation to job characteristics such as perceived meaningfulness of the work or job to the employee.

The current study established that mental retirement has a steady structure in a broad population and across different subgroups. The concept of mental retirement may become more important since sustainable employability is becoming a more essential and important topic for individuals, organizations as well as society. The life expectancy of people is getting higher due to lower birthrates as well as a decline in mortality rates, and the official pension age is increasing too [19, 37]. This is reflected in the rapid ageing of the working population [19]. The effective retirement age has increased considerably in the past decade in the OECD countries (Organisation for Economic Co-operation and Development): from 63.3 years for males and 61.3 years for females in 2002 to respectively 64.2 and 63.1 years in 2012 [19]. Therefore, there is an increasing pressure for maintenance of physical, mental and cognitive abilities of the labor force. The prevention of mental retirement can play an important role in the maintenance of these abilities.

When interpreting the findings of this study, some limitations should be kept in mind. First, this article focuses only on the structure of mental retirement and did not involve possible determinants or effects of mental retirement. This was not the aim of the present study and did not fit the data structure used for the purpose of this study. This study only tested the construct validity and no antecedent nor outcome variables were included. The causal chain of antecedent variables to mental retirement and its outcomes should preferably be studied in a longitudinal design. Secondly, one of the subcomponents of mental retirement, perceived appreciation, was measured with only 1 item and therefore the reliability and validity of this component is limited. Thirdly, although this study tested the construct validity, more validity testing, such as content, convergent and predictive validity, is warranted to strengthen the concept of mental retirement. Thus, there is a further need for replication of studies on the concept of mental retirement. Future research should be longitudinal in nature and study factors that may cause mental retirement, as well as relevant outcomes of this state. These studies could also focus on interventions that impact on ‘developmental proactivity’, ‘work engagement’ and ‘perceived appreciation’ in preventing or decreasing the level of mental retirement in organizations.

## Conclusions

This study clearly shows that the three-factor model of mental retirement, which consists of developmental proactivity, work engagement and perceived appreciation, is confirmed in a general group of employees as well as across different subgroups of age, level of education and occupation. Since sustainable employability is more and more essential in today’s society, there is an increasing pressure for maintenance of physical, mental and cognitive abilities of the labor force. The prevention of mental retirement may play an important role in the maintenance of these abilities.

## Data Availability

The datasets generated during and/or analyzed during the current study are not publicly available due to the privacy of the participants, but are available from the corresponding author on reasonable request.

## Appendix

**Table 7:** Questionnaire of mental retirement

Items of mental retirement	
Developmental proactivity	
1	In my work, I keep trying to learn new things.
2	I think about how I can keep doing a good job in the future.
3	In my work, I search for people from whom I can learn something.
4	With regard to my skills and knowledge, I see to it that I can cope with changes in my work.
Work engagement	
1	At my work, I feel bursting with energy.
2	At my job, I feel strong and vigorous.
3	I am enthusiastic about my job.
4	My job inspires me.
5	When I get up in the morning, I feel like going to work.
6	I am proud on the work that I do.
Perceived appreciation	
1	Do you feel appreciated in your current job?

## Abbreviatons

CFA: confirmatory factor analysis
UWES: Utrecht work engagement scale
RMSEA: Root mean square error of approximation
SRMR: standardized root mean square residual
TLI: Tucker-lewis index
CFI: Comparative fit index
OECD: Organisation for economic co-operation and development
